# Anticancer Properties of Carnosol: A Summary of In Vitro and In Vivo Evidence

**DOI:** 10.3390/antiox9100961

**Published:** 2020-10-08

**Authors:** Eric J. O’Neill, Danja J. Den Hartogh, Karim Azizi, Evangelia Tsiani

**Affiliations:** 1 Department of Health Sciences, Brock University, St. Catharines, ON L2S 3A1 Canada; eo15nv@brocku.ca (E.J.O.); dd11qv@brocku.ca (D.J.D.H.); ka14qr@brocku.ca (K.A.); 2 Centre for Bone and Muscle Health, Brock University, St. Catharines, ON L2S 3A1, Canada

**Keywords:** cancer, survival, proliferation, apoptosis, carnosol, in vitro, in vivo

## Abstract

Cancer is characterized by unrestricted cell proliferation, inhibition of apoptosis, enhanced invasion and migration, and deregulation of signalling cascades. These properties lead to uncontrolled growth, enhanced survival, and the formation of tumours. Carnosol, a naturally occurring phyto-polyphenol (diterpene) found in rosemary, has been studied for its extensive antioxidant, anti-inflammatory, and anticancer effects. In cancer cells, carnosol has been demonstrated to inhibit cell proliferation and survival, reduce migration and invasion, and significantly enhance apoptosis. These anticancer effects of carnosol are mediated by the inhibition of several signalling molecules including extracellular signal-regulated kinase (ERK), p38, c-Jun *N*-terminal kinase (JNK), Akt, mechanistic target of rapamycin (mTOR) and cyclooxygenase-2 (COX-2). Additionally, carnosol prevents the nuclear translocation of nuclear factor kappa-light-chain-enhancer of activated B cells (NF-κB) and promotes apoptosis, as indicated by increased levels of cleaved caspase-3, -8, -9, increased levels of the pro-apoptotic marker Bcl-2-associated X (BAX), and reduced levels of the anti-apoptotic marker B-cell lymphoma 2 (Bcl-2). The current review summarizes the existing in vitro and in vivo evidence examining the anticancer effects of carnosol across various tissues.

## 1. Introduction

Cancer is characterized by the unregulated proliferation of cells, the inhibition of programmed cell death—known as apoptosis—altered metabolism, tissue invasion and metastasis, and dysregulation of cell signalling that ultimately leads to enhanced survival, growth, and tumour formation [1]. Mutations and epigenetic changes allow cancer cells to grow and replicate uncontrollably and invade normal tissues [2]. This uncontrolled growth and proliferation arises due to loss of function mutations in tumour suppressor genes such as *TP53* which encodes the tumour suppressor protein p53, as well as, mutations converting proto-oncogenes to oncogenes which lead to the hyperactivation of signalling pathways that promote growth and survival such as the PI3K-Akt pathway and the Ras-ERK pathway [2].

Despite the established treatments of cancer that include surgery, chemotherapy, and radiotherapy, cancer-related deaths are on the rise globally [3]. Novel plant-derived chemicals may provide an alternate effective method for the treatment of cancer. For much of history, people have used plants and plant extracts to treat their ailments [4]. Outside the Western world, phytotherapy still remains very common, and has since gained popularity in the West, with an estimated 30% of Americans currently using plant-based remedies [5]. Furthermore, many established pharmaceuticals are derived from plants, such as metformin, which was isolated from French lilac, morphine from the opium poppy, and aspirin from willow tree bark [6]. Developing an understanding of plant extracts and plant-derived compounds continues to provide great opportunities to assess and establish new chemicals for preventative and therapeutic purposes.

Polyphenols are a class of compounds found abundantly in many plant species that are regularly consumed by humans [7]. These compounds are partially responsible for the colour, fragrance, and taste of many fruits, vegetables, and herbs. Many polyphenols possess a bitter, astringent flavour often associated with foods such as nuts, tea, coffee, cider, and wine [8]. However, the properties of polyphenols go beyond just the taste and smell of food as they have been found to possess significant antioxidant, anti-inflammatory, cardioprotective, neuroprotective, antidiabetic, and anticancer properties [9,10,11,12]. Therefore, the study of natural products and plant-derived polyphenols is important as it provides opportunities to uncover the therapeutic potential of compounds people may already be consuming on a regular basis.

**Figure 1:**
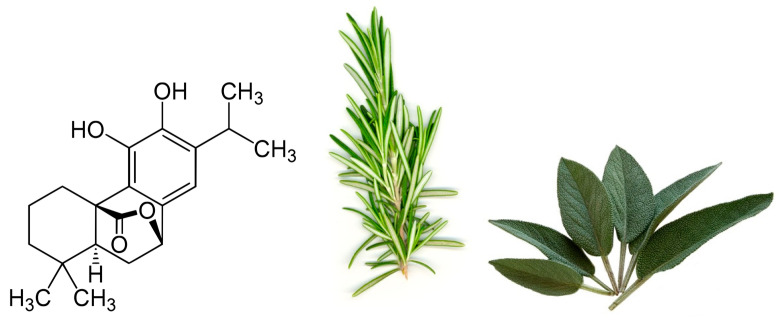
Chemical structure of carnosol found in rosemary and sage.

One particular polyphenol of interest is carnosol. Carnosol (Figure 1) is an ortho-diphenolic diterpene that contains an abietane carbon skeleton with hydroxyl groups at positions C-11 and C-12 and a lactone moiety across the B ring [13]. It is a naturally occurring polyphenol that is produced by the oxidative degradation of carnosic acid and is found in many herbs including rosemary (*Rosmarinus officinalis*) and sage (*Salvia officinalis*; Figure 1) [14,15,16]. The extracts of these plants have been demonstrated to have antioxidant, antimicrobial, antidiabetic, and anticancer properties with minimal toxicity [17,18,19,20]. Carnosol has been identified as a major component of these extracts which has led to research on the effects this individual compound has on various models of disease.

Carnosol has been proven to have potent antioxidant effects in cell-free, in vitro cell culture, and in vivo animal models. The accumulation of reactive oxygen species (ROS), formed through normal cellular functions as well as environmental exposure, can lead to the oxidation of DNA and proteins, which contributes to the development of diseases such as cancer [21,22]. Antioxidants can protect cells from ROS-induced damage by directly scavenging free radicals and/or activating endogenous enzymes and molecules that promote redox homeostasis [22,23]. In cell-free experiments, carnosol was able to inhibit lipid peroxidation, scavenge peroxyl radicals, protect anti-proteinase from hypochlorous acid-induced inactivation, scavenge hydroxyl radicals, and reduce cytochrome c, therefore confirming carnosol as a potent antioxidant [24]. Conversely, carnosol promoted bleomycin/iron-induced DNA damage indicating an ability to act as a pro-oxidant under certain conditions [24].

In vitro studies have demonstrated the nuclear factor erythroid derived 2-related factor 2 (Nrf2) to be involved in the cytoprotective effect of carnosol [25,26]. Treatment of HepG2 cells with 5 µM carnosol for 12 h exhibited a cytoprotective effect against both hydrogen peroxide and alcohol and resulted in a 160% increase in levels of glutathione (GSH), a tripeptide involved in the detoxification of chemical substances, with no adverse effect on cell viability [26]. Additionally, carnosol treatment led to increased levels of Nrf2 in the nucleus and Nrf2 knockdown via siRNA abolished the carnosol-induced increase of GSH synthesis enzymes [26]. Carnosol also supressed tumour necrosis factor alpha (TNF-α)-induced nuclear translocation of NF-κB, but this effect was abolished when carnosol treatment was performed alongside buthionine sulfoximine, a GSH synthesis blocker [26]. Another study found that carnosol (5–20 µM) treatment of microglia cells for 6 and 24 h was able to induce Nrf2 as well as heme oxygenase-1, an enzyme involved in counteracting oxidative stress, with minimal cytotoxicity [25]. These antioxidant properties of carnosol have also been confirmed in animal models where intraperitoneal injection of carnosol (100–400 mg/kg) in female Sprague–Dawley rats led to a 1.6- to 1.9-fold increase in glutathione-S-transferase (GST) activity and a 3.1- to 4.8-fold increase in NAD(P)H-quinone reductase (QR) activity, two enzymes involved in detoxification of chemical substances [27]. In summary, these studies show carnosol to exhibit antioxidant properties by directly scavenging free radicals as well as inducing cellular pathways that counteract oxidative stress. Studies provide evidence that many established cancer treatments such as paclitaxel increase ROS levels in cancer cells resulting in cytotoxic/cancer killing effects [28]. Therefore, more studies are required to investigate whether carnosol acts as a pro-oxidant or antioxidant in cancer cells in vitro and in clinical studies.

Although many studies provided evidence of the beneficial effects of carnosol against different diseases, only a limited number of studies have examined its bioavailability and pharmacokinetics [29,30,31,32]. Oral administration of 100 mg of rosemary extract (RE) enriched with carnosic acid to lean female Zucker rats produced a plasma carnosol concentration of 18.2 µM [31]. Furthermore, oral administration of RE resulted in carnosol being detected in tissues of the stomach, duodenum, jejunum, ileum, and liver, and only present in trace amounts in the brain [30,31]. These studies indicate that oral administration of rosemary extract, which contains carnosol, can result in blood carnosol levels in the micromolar range and significant levels in different tissues in the body. Many in vitro studies have shown that carnosol at micromolar levels can influence various biological functions and modify key signalling pathways [30,31,32]. Unfortunately, no animal studies exist where carnosol was administered orally or intraperitonially to animals followed by measurements of blood carnosol levels. In addition, no studies exist investigating the bioavailability and metabolism of carnosol in humans. Carnosol and polyphenols in general are hydrophobic and have low intestinal absorption rate. To bypass these absorption problem researchers are experimenting with encapsulation approaches but we have not found any such studies for carnosol as is the case for polyphenols such as resveratrol [33] and curcumin [34]. A study by Soler-Rivas et al. (2010) showed that carnosol present in a supercritical fluid extract of rosemary in sunflower oil (42 mg RE/g) is 62.59% bioaccessible to target tissues and increases to 87.85% by the addition of lecithin (37 mg/g) [29].

The current review summarizes the existing studies examining the effects of carnosol on in vitro and in vivo models of cancer. Cancers of the stomach, liver, rectum, oesophagus, cervix, and thyroid rank in the top ten in terms of cancer incidence globally; however, no studies on the effects of carnosol on these tissues were identified [3]. Additionally, no in vivo studies on cancers of the lung, colon, pancreas, leukemia, or brain were identified. The studies are presented chronologically and grouped by tissue/organ in order of mortality rate: lung, colon, breast, pancreas, prostate, leukemia, brain, and skin [3]. 

## 2. Literature Review

### 2.1. Effects of Carnosol on Lung Cancer In Vitro

Lung cancer is the most commonly diagnosed form of cancer as well as the most fatal [3]. There are two main subclasses of lung cancer, non-small-cell lung cancer and small-cell lung cancer, with the former accounting for approximately 85% of cases [35]. Non-small-cell lung cancer is further divided based on histology into squamous-cell carcinoma, adenocarcinoma, or large-cell lung cancer [35]. Earlier stages of non-small-cell lung cancer are typically treated with surgery while more advanced local stages are treated with a regimen of radiation therapy and chemotherapy followed by surgery [36].

A study by Offord et al. (1995) showed that carnosol can attenuate polycyclic aromatic hydrocarbon (PAH)-induced carcinogenesis in BEAS-2B human bronchial cells. Benzo[a]pyrene (B[a]P) is a carcinogenic PAH whose metabolites induce mutations through the formation of DNA adducts [37]. Pretreatment with carnosol (0.6–6 µg/mL) for 1 h followed by cotreatment with B[a]P for an additional 6 h caused dose-dependent inhibition of B[a]P-induced DNA adduct formation. Six-h treatment with B[a]P alone caused a ten-fold increase in cytochrome P540-1A1 (CYP1A1) mRNA expression determined using the RNase protection technique. Carnosol (1 µg/mL) pretreatment for 1 h followed by cotreatment with B[a]P for 6 h reduced this B[a]P-induced increase in CYP1A1 mRNA by half [37]. Furthermore, carnosol (1 µg/mL) pretreatment for 1 h followed by cotreatment with B[a]P for 6 more hours caused an 80% reduction in the activity of CYP1A1 as measured using an ethoxyresorufin-O-deethylase (EROD) assay. Interestingly, preincubation with carnosol before the addition of B[a]P was required to reduce CYP1A1 activity and the later carnosol was added after B[a]P, the less carnosol was able to reduce B[a]P-induced CYP1A1 activity [37]. In addition, the effects of carnosol on GST, an enzyme that detoxifies carcinogenic B[a]P metabolites by conjugating them with glutathione, were investigated. Carnosol (1 µg/mL) treatment for 16 to 24 h caused a three- to four-fold increase in GSTπ mRNA expression with a 50–100% increase in GSTπ protein expression, but no effect on GST activity. The same treatment also produced an increase in QR mRNA (Table 1) [37]. Overall, these results indicate that carnosol may act as a chemopreventative agent through the inhibition of CYP1A1 and the upregulation of detoxifying enzymes: GST and QR.

### 2.2. Effects of Carnosol on Colon Cancer In Vitro

Colon cancer is the fourth most common cancer diagnosis globally; however, it disproportionately affects Westernized countries [3,38]. This discrepancy in the incidence of colon cancer between cultures may be due to differences in diet where the Western diet typically consists of more fat and less dietary fibre [38]. Colon cancer is typically treated with surgery, or various combinations of surgery, targeted radiation therapy, and systemic chemotherapy may also be used [39].

Park et al. (2014) showed that treatment of HCT116 human colon cancer cells with carnosol (5–100 µM) for 24–72 h produced a dose- and time-dependent reduction in cell viability [40]. Analysis of cell morphology using fluorescence microscopy showed evidence of carnosol-induced apoptosis and this result was confirmed with fluorescein isothiocyanate (FITC)-annexin V/propidium iodide staining and flow cytometry. Additionally, carnosol treatment produced a dose-dependent increase in the levels of cleaved caspase-3, caspase-9, and poly (ADP-ribose) polymerase (PARP) as assessed with western blotting and increased caspase-3 activity measured using a colorimetric caspase-3 activity assay [40]. Treatment of cells with the ROS scavenger *N*-acetyl-L-cysteine (NAC) abolished the carnosol-induced increase in cleaved PARP and caspase-3, indicating the involvement of ROS in carnosol-induced apoptosis [40]. Furthermore, western blotting analysis showed that carnosol produced a dose-dependent increase in p53 and a corresponding reduction in mouse double minute 2 homolog (Mdm2), which promotes the proteasomal degradation of p53, as well as a dose-dependent increase in pro-apoptotic BAX expression with a concomitant decrease in anti-apoptotic Bcl-2 expression [40]. Carnosol treatment was also found to inhibit constitutive phosphorylation (Tyr705) of signal transducer and activator of transcription (STAT) 3, reduce nuclear localization of phosphorylated STAT3, and reduce STAT3 DNA-binding activity. Moreover, carnosol treatment of HCT116 cells transfected with a STAT3-luc construct saw a reduction in STAT3 reporter gene activity. These reductions in STAT3 corresponded to attenuated expression of cyclin-D1, -D2, -D3, and survivin which are all products of STAT3 target genes [40]. Lastly, Park et al. (2014) found carnosol treatment to reduce the phosphorylation of Jak2 and Src, two upstream kinases that lead to the phosphorylation of STAT3. Treatment of cells with AG490 and PP2 (inhibitors of Jak2 and Src) confirmed that reduced phosphorylation of Jak2 and Src corresponds with reduced phosphorylation of STAT3 (Table 2) [40]. Ultimately, these results indicate that carnosol-induced apoptosis in colon cancer cells is mediated through ROS generation, the activation of caspases, the induction of p53, and STAT3 inhibition.

### 2.3. Effects of Carnosol on Breast Cancer In Vitro

Breast cancer is the most common form of cancer in women, accounting for about one quarter of female cancer diagnoses [41]. Breast cancer is categorized into three major subtypes depending on the presence of estrogen or progesterone hormone receptors and the presence of human epidermal growth factor receptor 2 (HER2). Hormone receptor-positive breast cancers are typically treated with endocrine therapy, HER2-positive breast cancers are typically treated with targeted antibody or small-molecule inhibitor therapy combined with chemotherapy, and triple-negative breast cancers are usually treated with chemotherapy alone [42]. Of the three breast cancer subtypes, triple-negative is the most fatal and most complicated to treat and the median overall survival of metastatic patients is only 1 year [42]. 

In a study by Subbaramaiah et al. (2002), treatment of 184B5/HER breast cancer cells with carnosol (20–60 μM) for 8 h resulted in a significant reduction in the expression of COX-2, an enzyme often overexpressed in cancer cells that catalyzes the synthesis of prostaglandins form arachidonic acid [43]. Transcription of *COX-2* is induced by phorbol ester (PMA) through increased binding of AP-1 to the cyclic AMP response element (CRE) of the *COX-2* promoter [43]. Treatment of 184B5/HER cells with carnosol (60 µM) for 8 h inhibited PMA-mediated AP-1 binding to the CRE of the *COX-2* promoter. Carnosol was also found to reduce PMA- and EGF-induced increases in prostaglandin-2 (PGE_2_) mRNA levels [43]. Additionally, carnosol inhibited the activation of protein kinase C (PKC), ERK1/2, p38, and JNK (Table 3). Overexpression of JNK was able to reverse the effect of carnosol on *COX-2* transcription [43]. Ultimately, these results indicate that carnosol blocks JNK and PKC signalling, which, in turn, blocks the binding of AP-1 to the CRE of the *COX-2* promoter, leading to the suppression of COX-2 expression. 

Johnson et al. (2010) demonstrated that treatment of MCF7 breast cancer cells with 20–40 µM carnosol for 48 h led to decreased androgen receptor (AR) and estrogen receptor (ER-α) mRNA and protein levels, as well as a dose-dependent decrease in cell viability with a half maximal inhibitory concentration (IC_50_) value of 25.6 µM (Table 3) [44]. Additionally, carnosol induced morphological changes in the cells. 

Rodriguez and Potter (2013) used small interfering RNA (siRNA) in MCF7 and MDA-MB-231 breast cancer cells to knockdown CYP1A1 in order to investigate the role of this enzyme in breast cancer progression [45]. Knockdown of CYP1A1 led to decreased colony formation, decreased cell proliferation, G_0_-G_1_ cell cycle arrest, and increased AMP-activated protein kinase (AMPK) phosphorylation. Carnosol treatment was used to determine if the same outcomes can be achieved through pharmacological reduction in CYP1A1. Carnosol treatment (40 µM) for 8 h was shown to have a similar effect on breast cancer cells as CYP1A1 knockdown via siRNA [45]. Treatment with carnosol resulted in decreased proliferation, reduced survival, and increased apoptosis with IC_50_ values of approximately 40 µM for both cell lines. Additionally, carnosol increased phosphorylation of AMPK and reduced expression of the aryl hydrocarbon receptor (AhR), the substrates of which induce expression of CYP1A1, by more than half (Table 3) [45]. Knockdown of AhR via siRNA had no effect on the carnosol dose–response curve indicating that the antiproliferative effects of carnosol are independent of AhR [45]. Overall, these data show that carnosol impairs cancer cell proliferation and promotes apoptosis with a mechanism of action that involves AMPK activation and a reduction in CYP1A1. 

Another study found treatment of triple-negative MDA-MB-231 breast cancer cells with carnosol (25–100 μM) for 24–48 h significantly reduced cellular viability, and induced G2/M cell cycle arrest both dose- and time-dependently [46]. Treatment with carnosol (50–100 μM) resulted in increased protein levels for markers of cell cycle arrest (p21) and apoptosis (cleaved PARP and caspase-3, -8, and-9) [46]. Additionally, carnosol treatment increased autophagy, as observed with light and electron microscopy, as well as increased microtubule-associated protein 1A/1B-light chain 3-phosphatidylethanolamine conjugate (LC3II) and decreased p62 protein levels. Knockdown of the autophagy effector Beclin1 with siRNA had no effect on carnosol-induced changes in markers of autophagy (LC3II and P62) indicating that the carnosol-induced autophagy is independent of Beclin1 [46]. Furthermore, carnosol treatment induced dose- and time-dependent increases in ROS as well as γH2AX protein levels, a marker of DNA damage. Scavenging of ROS with tiron abolished the carnosol-induced DNA damage, indicating that carnosol-induced DNA damage is a result of ROS (Table 3) [46].

In a study by Vergara et al. (2014), the treatment of breast cancer cell lines (HBL-100, MDA 231, 361, 435 and MCF-7) with carnosol (25–200 μM) for 24–72 h significantly reduced cell viability and cell adhesion [47]. Treatment with carnosol (12.5, 25 and 50 μM) resulted in 10, 50 and 75% reductions in cell adhesion to fibronectin-coated plates, respectively. The IC_50_ value for human breast cancer cell lines was greater than 50 μM and no effect was observed with carnosol treatment under 25 μM (Table 3) [47].

In a study by Telang et al. (2018), treatment of HER-2 overexpressing tumorigenic human mammary epithelial cells (184–B5/HER) with carnosol (1–5 μM) for 21 days dose-dependently inhibited colony formation and increased G2/M phase arrest [48]. Additionally, G2 phase specific cyclin B1 expression was upregulated with carnosol treatment compared to control cells (Table 3) [48].

Treatment of several breast cancer cell lines (MDA-MB-231, Hs578T, MCF-7, and T47D) with carnosol (25–100 μM) for 24 h reduced capacity for metastasis [49]. A wound-healing assay and Matrigel invasion assay showed that treatment with 25 µM carnosol was able to significantly inhibit cell migration and invasion. Matrix metalloproteinases (MMP) promote metastasis by facilitating the breakdown of extracellular matrix proteins, which allows for cell invasion. Treatment with carnosol dose-dependently reduced both the activity and secretion of MMP-9 and RT-PCR showed that this reduction correlated with a reduction in mRNA expression of MMP-9 [49]. Additionally, carnosol significantly reduced both total and phosphorylated STAT3 protein levels; however, carnosol had no effect on the level of STAT3 mRNA transcripts (Table 3). Furthermore, inhibition of autophagy with 3-methyladenine or chloroquine was unable to restore the carnosol-induced reduction in STAT-3 protein levels. Conversely, pre-treatment with proteasome inhibitors (MG-132 and bortezomib) abolished the carnosol-induced reduction in STAT3 protein indicating that carnosol targets STAT3 for proteasome degradation. Pre-treating cells with the ROS scavenger *N*-acetyl-L-cysteine (NAC) prevented the proteasomal degradation of STAT3, suggesting that carnosol targets STAT3 for degradation via a ROS-mediated mechanism [49]. 

Overall, these studies provide evidence that carnosol is effective in reducing the viability and proliferation of breast cancer cells. 

### 2.4. Effects of Carnosol on Breast Cancer In Vivo

Singletary et al. (1996) found that carnosol was able to inhibit tumorigenesis and the formation of 7,12-dimethylbenz[a]anthracene (DMBA)-DNA adducts in female Sprague–Dawley rats [50]. Dietary supplementation with carnosol (0.5% by weight) for two weeks had no effect on DMBA-induced mammary tumorigenesis; however, intraperitoneal injection of carnosol for 5 days at 200 mg/kg body weight inhibited mammary DMBA-DNA adduct formation by 40%. This inhibition of DMBA-DNA adduct formation corresponded to a 65% decrease in the number of DMBA-induced mammary adenocarcinomas (Table 4) [50]. Overall, these data indicate that intraperitoneal injections of carnosol can reduce the formation of mammary adenocarcinomas in rats.

Alsamri et al. (2019) inoculated chick embryos with triple-negative MDA-MB-231 breast cancer cells and then investigated the effects of carnosol treatment on the resulting tumour [49]. Ten-day old embryos were inoculated with 10^6^ MDA-MB-231/green fluorescent protein (GFP) cells onto the chorioallantoic membrane. Embryos were then treated the next day and every two days over the course of one week by dropping the respective treatment directly onto the tumour. Carnosol treatment at 50 µM and 100 µM was found to inhibit tumour growth by 65% and 75%, respectively [49]. Additionally, carnosol treatment was found to reduce metastasis with an average of 0.7 nodules distal to the site where breast cancer cells were implanted, compared to an average of six nodules in the control group (Table 4) [49]. Overall, these data demonstrate carnosol to be effective in reducing the growth and metastasis of tumours.

### 2.5. Effects of Carnosol on Pancreatic Cancer In Vitro

Pancreatic cancer is one of the deadliest forms of cancer, with a median survival of 3 months and a 5-year survival rate ranging from 2–9% [51,52]. It is the thirteenth most common cancer diagnosis, accounting for 4.5% of cancer deaths globally [3]. Surgical resection is currently the most viable treatment option for pancreatic cancer and is typically combined with chemotherapy [52]. The poor prognosis for pancreatic cancer highlights the importance and need for investigating novel treatment compounds.

Aliebrahimi et al. (2018) investigated the ability of carnosol to act as an inhibitor of c-Met in pancreatic ductal adenocarcinoma (PDAC) cells [53]. c-Met is a receptor tyrosine kinase that is activated by the ligand hepatocyte growth factor (HGF); however, abnormal activation of c-Met is responsible for the low survival rate and high relapse rate of PDAC. Treatment of the human PDAC cell line AsPC-1 with 100 ng/mL of HGF induced cancer cell proliferation. Treatment with carnosol (0–60.5 µM) for 48 h significantly inhibited HGF-dependent growth with an IC_50_ value of 14.56 µM [53]. Interestingly, the IC_50_ value was higher in HGF-free media, indicating that the inhibitory effects of carnosol are tied to the HGF/c-Met signalling pathway. Flow cytometry analysis found carnosol, at its IC_50_ concentration, to arrest AsPC-1 cells in the S phase of the cell cycle when compared to untreated cells. Furthermore, FITC-annexin V/propidium iodide staining and flow cytometry found 15 µM carnosol increased the proportion of cells in early apoptosis from 3% in the control group to 32% [53]. Additionally, 100 ng/mL HGF was found to promote 70% wound closure after 24 h in a scratch assay, but treatment with carnosol dose-dependently supressed this HGF-induced migration [53]. With regards to HGF-mediated c-Met signalling, carnosol only inhibited phosphorylated c-Met and Akt at high concentrations (50 and 75 µM), while lower concentrations of carnosol (10 and 25 µM) increased phosphorylated c-Met [53]. Moreover, carnosol (15 µM) reduced the self-renewal capacity of AsPC-1 cells, as determined by preventing the sphere growth of cells in suspension and decreasing colony formation in adherent cells from 48 colonies in the control to 18. Gene expression studies showed carnosol treatment downregulated the expression of oct-4 and nanog mRNA, genes implicated in the stemness properties of cancer stem-like cells (CSCs), with no significant effect on c-Myc mRNA (Table 5) [53]. Overall, these data indicate that carnosol can act as a c-Met inhibitor to reduce HGF-driven proliferation and migration in PDAC cells, as well as reducing their ability to form pancreatic CSCs.

### 2.6. Effects of Carnosol on Prostate Cancer In Vitro

Prostate cancer is the fifth leading cause of death globally and the second most common cancer diagnosis in men [54]. Less severe forms of the disease can be managed via active surveillance while more severe or advanced stages of prostate cancer are typically treated with surgery and radiation therapy [55]. 

Treatment of androgen-insensitive PC3 prostate cancer cells with carnosol (10–70 μM) for 24–72 h significantly reduced cell viability and increased apoptosis both dose- and time-dependently (Table 6) [56]. Carnosol led to increased G2 phase cell cycle arrest as well as increased pro-apoptotic phosphatase and tensin (PTEN), eukaryotic translation initiation factor 4E-binding protein 1 (4EBP1), caspase-7, caspase-8, and BAX protein levels. Conversely, carnosol treatment led to a reduction in the protein expression of anti-apoptotic and antiproliferation Bcl-2, p21 and both the p85 and p110 subunits of phosphatidylinositol 3-kinase (PI3K) [56]. Additionally, carnosol treatment saw the upregulation of the AMPK-β1 regulatory subunit and a dose-dependent decrease in Thr172 phosphorylation of the catalytic AMPK-ɑ subunit. This decrease in AMPK phosphorylation coincided with a decrease in the phosphorylation of downstream targets of AMPK: mTOR and p70 S6K [56]. These data suggest that carnosol treatment reduces anti-apoptotic and antiproliferative protein levels and increases pro-apoptotic protein levels.

Treatment of LNCaP and 22Rv1 prostate cancer cells with carnosol (20–40 µM) for 48 h led to decreased expression of AR and ER-α mRNA and protein levels, as well as dose-dependently decreased cell viability with IC_50_ values of 19.6 µM and 22.9 µM in LNCaP and 22Rv1 cells, respectively (Table 6) [44]. Additionally, the carnosol-induced decrease in viability was accompanied by a change in cell morphology as observed with light microscopy. Moreover, carnosol was found to bind to AR and ER-α in a cell-free assay. Using a cell-based assay with AR-UAS-bla GripTite 293 cells and ER-α-UAS-bla GripTite 293 cells, it was determined that carnosol does not act as an agonist for either receptor, but instead acts as an antagonist for both AR and ER-α [44]. Furthermore, immunofluorescence microscopy showed that carnosol led to decreased protein expression of AR and ER-α as well as inhibited nuclear translocation of these receptors in LNCaP cells. Treatment of LNCaP cells with the antagonists flutamide or tamoxifen caused an increase in AR and ER-α protein levels respectively; however, cotreatment with carnosol abolished this increase, suggesting that combination treatments with carnosol may be beneficial [44].

Treatment of hormone-dependent LNCaP prostate cancer cells and hormone-independent DU145 prostate cancer cells with carnosol (0.25–16 μM) for 48 h significantly reduced cell survival and increased apoptosis [57]. LNCaP cells had upregulation of both mRNA and protein levels for the tumorigenesis transcription factors glioma-associated oncogene homolog 1 (Gli1) and Sonic hedgehog (Shh). Carnosol treatment led to a dose-dependent reduction in Gli1 and Shh mRNA and protein with a greater inhibitory effect observed in the hormone-dependent LNCaP cells. Additionally, carnosol led to a dose-dependent increase in caspase-3 activity suggesting carnosol may promote apoptosis [57]. Overall, these data indicate that carnosol can reduce proliferation and induce apoptosis via a mechanism likely associated with inhibition of hedgehog signalling (Table 6).

### 2.7. Effects of Carnosol on Prostate Cancer In Vivo

Oral administration of carnosol (30 mg/kg/day) 5 days per week for 4 weeks to athymic nude mice implanted with AR and ER-α positive 22Rv1 cells reduced tumour growth by 36% and reduced serum prostate specific antigen levels by 26% [44]. Additionally, western blot analysis of tumours showed a decrease in AR and ER-α protein expression in the carnosol-treated group (Table 6) [44]. Overall, these data indicate that oral administration of carnosol can reduce cancer progression in xenograft models of prostate cancer.

### 2.8. Effects of Carnosol on Leukemia In Vitro

The leukemias are blood cancers caused by malignant transformations in hematopoietic stem or progenitor cells of the bone marrow, lymph nodes, or other lymphoid tissue [58]. Leukemias are broadly categorized as acute or chronic and myeloid or lymphoblastic leukemia with many more subtypes within these categories [58]. Acute lymphoblastic leukemia (ALL) is the most common cancer diagnosis in children and the most common cause of cancer-related deaths in people under 20 years of age [59]. An extensive treatment plan involving multiple phases of chemotherapy is required to treat ALL; however, this produces severe toxic effects such as the development of osteonecrosis which usually requires surgical management [59]. The potential negative side effects of current treatment options underline the need for novel approaches to treat leukemia. 

Dörrie et al. (2001) showed that treatment with carnosol induced apoptosis and downregulation of Bcl-2 in several B-lineage leukemia cell lines [60]. Treatment of several B-lineage ALL-derived cell lines (SEM, RS4;11, MV4;11, REH, and Nalm-6) with carnosol (9–27 µM) resulted in a dose-dependent reduction in cell viability. The same carnosol treatments were applied to peripheral blood mononuclear cells isolated from healthy individuals and it was determined that carnosol concentrations of 18 µM or less had no significant effect on the viability of non-cancerous cells [60]. Furthermore, treatment of ALL-derived cell lines with 18 µM carnosol for 24 h resulted in a significant number of cells in early apoptosis, as determined via FITC-annexin V/propidium iodide staining and flow cytometry. Additionally, flow cytometry with JC-1 dye showed that carnosol treatment increased the proportion of cells with depolarized mitochondrial membranes [60]. Lastly, flow cytometry was used to examine Bcl-2 expression in the remaining viable cells after carnosol treatment. All five ALL-derived cell lines exhibited a reduction in Bcl-2 expression compared to untreated cells and, since this was observed in viable cells, it suggests that carnosol-induced downregulation of Bcl-2 occurs at an early stage before any obvious changes associated with apoptosis (Table 7) [60]. Overall, these data suggest that carnosol can promote apoptosis by inducing the downregulation of Bcl-2.

Ishida et al. (2014) investigated the ability of carnosol to induce apoptosis in adult T-cell leukemia/lymphoma (ATL) [61]. Firstly, treatment of ED cells, an ATL cell line, with 40 µM carnosol was shown to increase apoptosis, as indicated by FITC-annexin V/propidium iodide staining and flow cytometry, as well as the increase activated caspase-3 and caspase-7 protein levels [61]. Two-dimensional differential gel electrophoresis and mass spectrometry revealed that treatment with 40 µM carnosol for 24 h led to the increased expression of nicotinamide adenine dinucleotide phosphate (NADPH)-dependent reductases, glycolytic enzymes, and enzymes in the pentose phosphate pathway. These results were confirmed with western blotting analysis, which showed that treatment with carnosol increased the expression of moesin, annexin A1, α-enolase, and thioredoxin reductase (Table 7). This increased expression of NADPH-dependent reductases and upregulation of pathways that contribute to the production of NADPH suggest that the action of carnosol is related to NADPH-dependent redox regulation in the cells [61]. Next, Ishida et al. (2014) examined how carnosol affects glutathione, an antioxidant that protects cells against oxidative stress by cycling between its reduced state (GSH) and its oxidized state (GSSG). Treatment of ED cells with 40 µM carnosol for 3 and 6 h saw a decrease in intracellular GSH and GSSG levels with no change in the GSH/GSSG ratio. Exogenous supplementation of carnosol-treated cells with NAC, a precursor to glutathione, restored cell viability, confirming the relationship between carnosol-induced apoptosis and glutathione depletion [61]. Lastly, treating the cells with carnosol in media containing catalase, an H_2_O_2_ scavenger, did not reduce the effect of carnosol confirming that carnosol’s action is not simply due to a polyphenol-induced increase in ROS in the cell culture media, which sometimes occurs with other polyphenols [61,62,63]. Ultimately, these results indicate that the ability of carnosol to induce apoptosis in ATL cells is caused by the depletion of glutathione. 

### 2.9. Effects of Carnosol on Brain Cancer In Vitro

Primary brain tumours are rare, accounting for only 1.6% of cancer diagnoses and 2.5% of cancer deaths; however, they are more common in young people, accounting for up to 20% of cancer deaths in people under 20 years of age [3,64,65]. Glial cell tumours (gliomas) are the most common central nervous system tumour, making up three quarters of cases, with glioblastoma being the most common and most fatal due to its 5-year survival rate of less than 3% [64,65]. Treatment for glioblastoma typically takes a multimodal approach consisting of surgical resection, wherever possible, followed by radiotherapy and chemotherapy; however, the median survival is still only 14–15 months [66,67]. This poor prognosis for brain cancer emphasises the need to explore new treatments.

A study by Giacomelli et al. (2016) investigated the effects of carnosol on three glioblastoma cell lines: U87MG, U343MG, and T98G [68]. The U87MG and U343MG cell lines express wild type p53 and overexpress MDM2, whereas T98G cells express a mutated p53 isoform. Carnosol treatment of U87MG cells caused a dose- and time-dependent reduction in proliferation with IC_50_ values of 28.9, 14.9, and 10.4 µM for 24-, 48-, and 72-h treatments, respectively. Washing out the carnosol treatment with drug-free media for 72 h was able to completely restore proliferation with low doses of carnosol (<20 µM) and partially restore proliferation with intermediate doses of carnosol (20–30 µM). However, higher concentrations of carnosol (>30 µM) irreversibly inhibited proliferation [68]. Interestingly, carnosol treatment of normal human mesenchymal stem cells caused a much less pronounced reduction in proliferation than in U87MG cells [68]. A dose- and time-dependent reduction in proliferation was also observed in U343MG cells with IC_50_ values of 19.0 and 11.7 µM for 48- and 72-h treatments, respectively. Performing the same treatments on T98G cells caused a significant right shift of the dose–response curve when compared to U87MG and U343MG cells suggesting that reactivation of the p53 pathway is implicated in the effects of carnosol; however, p53 knockdown via siRNA only partially inhibited the anti-proliferative action of carnosol [68]. Furthermore, treatment with carnosol resulted in a dose-dependent increase in p53 protein levels. The presence of the protein synthesis inhibitor CHX only slightly reduced the carnosol-induced increase in p53 protein, indicating that carnosol does not trigger de novo synthesis of p53. Using a direct quantitative sandwich immuno-enzymatic ELISA, it was determined that carnosol (1 µM) caused dissociation of the p53/MDM2 complex to 45.1% of control. These data were confirmed by a co-immunoprecipitation assay where a significant decrease in p53 was observed in MDM2 immunoprecipitates following carnosol treatment [68]. Additionally, carnosol treatment led to an increase in the transcription of p53-target genes: p21, PUMA, and MDM2. Increased transcription of the pro-apoptotic protein BAX and decreased transcription of the anti-apoptotic protein Bcl-2 was seen with carnosol treatment. Using flow cytometry, it was determined that carnosol induced apoptosis and caused G2-phase cell cycle arrest [68]. Moreover, a wound-healing assay showed the dose-dependent inhibition of cell migration by carnosol (Table 8). Lastly, Giacomelli et al. (2016) performed an isobolographic analysis to determine the combined effect of carnosol with temozolomide (TMZ), a common chemotherapeutic used in the treatment of glioblastoma. Carnosol was found to act synergistically with TMZ [68]. Overall, these data indicate that carnosol triggers the reactivation of p53 in human glioblastoma cell lines which ultimately leads to inhibition of proliferation and induction of apoptosis.

In another study, Giacomelli et al. (2017) found that carnosol modulates the epithelial–mesenchymal transition (EMT) and induces CSC apoptosis [69]. In order to identify how carnosol affects other features of cancer cells, carnosol concentrations less than the IC_50_ values for the inhibition of proliferation were chosen. Inflammatory priming with TNF-ɑ and transforming growth factor (TGF)-β1 was used to induce the EMT and was confirmed by a change in cell morphology from ovular to elongated and fibroblast-like, as well as an increase in the expression of the mesenchymal marker N-cadherin, alongside a decrease in the expression of the epithelial marker E-cadherin [69]. Treatment with 10 µM carnosol alone increased E-cadherin mRNA and protein levels whereas the same treatment did not change N-cadherin mRNA levels and decreased N-cadherin protein levels. Similarly, 10 µM carnosol was able to reduce the TNF-ɑ/TGF-β1-induced EMT, as indicated by the lower N-cadherin protein and mRNA, higher E-cadherin protein and mRNA, and cell morphology [69]. Carnosol alone had no effect on the expression of the transcription factors that regulate the EMT (Snail, Slug, Twist, and ZEB1); however, carnosol did attenuate the cytokine-induced up-regulation of Slug, Twist, and ZEB1 [69]. Treatment with carnosol attenuated the TNF-ɑ/TGF-β1-induced decrease in miR-200c, a miRNA that acts as a negative regulator of the EMT [69]. Moreover, 10 µM carnosol was able to decrease the expression of the stemness genes *CD44*, *Nanog*, *Oct4*, *BMI1* and *SOX2,* indicating a reduction in the fraction of CSCs in the population of U87MG cells. This reduction in stemness was further indicated by a dose-dependent reduction in number and diameter of neurospheres formed by U87MG cells as well as a decrease in mRNA expression of stem cell marker genes (*CD44, Nanog, Nestin,* and *OLIG2*) and increased expression of differentiated cell marker gene (*GFAP*) [69]. Carnosol treatment dose-dependently and irreversibly reduced the viability of CSCs derived from U87MG, U343MG, and T98G cells, although the effect was less pronounced in T98G cells, which express a mutated isoform of p53. In addition, treatment of U87MG-derived CSCs with carnosol increased mRNA expression of p53 target genes (*p21*, *PUMA*, and *MDM2*), increased expression of BAX, and decreased expression of Bcl-2, which was coupled with increased apoptosis and G2-phase cell cycle arrest, as determined by flow cytometry (Table 8) [69]. Lastly, carnosol was found to interfere with TNF-ɑ/TGF-β1-induced EMT in U87MG-derived CSCs in a similar way to wild type U87MG cells although the effect was less pronounced and required higher concentrations of carnosol [69]. Overall, these data indicate that carnosol not only reduces the viability of differentiated glioblastoma cells but can also inhibit some of the processes that contribute to the aggressive and resistive nature of glioblastoma, including EMT and CSC formation.

### 2.10. Effects of Carnosol on Skin Cancer In Vitro

Skin cancers are the most common type of cancer in Caucasians and the incidence of skin cancer is increasing globally [70,71]. Skin cancers are typically categorized as malignant melanoma or non-melanoma skin cancer which includes basal cell carcinoma and squamous cell carcinoma [72]. Non-melanoma skin cancer is the most common; however, malignant melanoma accounts for the majority of skin cancer-related deaths [72]. Natural polyphenols and other phytochemicals have been reported to have anti-cancer properties with regards to skin cancer [73,74].

Treatment of B16/F10 mouse melanoma cells with carnosol (1.25–20 μM) for 6–24 h resulted in dose- and time-dependent reductions in colony formation, cell migration, and invasiveness with 5 µM carnosol sufficient enough to inhibit colony formation by 93% [75]. While 10 µM carnosol treatment for 8 h was able to reduce invasive activity by 19%, 2.5–10 µM carnosol was only able to decrease viability by up to 10% and flow cytometry revealed no changes in cell cycle program compared to control [75]. Expression of MMP-2 and MMP-9 are higher in melanoma cells which helps facilitate metastasis. Gelatin zymography revealed carnosol to inhibit MMP-2 and MMP-9 activity in a dose-dependent manner with an IC_50_ value of approximately 5 µM. This decrease in activity correlated to a suppression in MMP-9 mRNA and protein expression with no effect on MMP-2 mRNA and protein levels; however, there was a slight reduction in protein expression of TIMP-2, an endogenous inhibitor of MMP-2 [75]. Furthermore, carnosol was found to inhibit the phosphorylation of AKT, p38, JNK, and ERK1/2 and had no effect on FAK, STAT1, or STAT3. Additionally, carnosol treatment led to reduced nuclear translocation of NF-κB and c-Jun. Carnosol was also found to inhibit the proteolytic degradation of IkB-ɑ, further confirming the inhibition of NF-κB activation since the activation of NF-κB correlates to the rapid proteolytic degradation of IkB-ɑ (Table 9) [75]. Overall, carnosol appears to inhibit invasion of B16/F10 cells by supressing MMP-9 via down-regulation of NF-κB and c-Jun.

Mohebati et al. (2012) investigated the effects of carnosol on PAH-induced carcinogenesis in HaCaT human keratinocytes [76]. Treatment of HaCaT cells with B[a]P, a PAH, activates AhR leading to increased expression of CYP1A1 and CYP1B1, whose respective proteins convert PAH to genotoxic metabolites. Treatment with carnosol (1–10 µM) for 2 h reduced these B[a]P-induced increases in CYP1A1 and CYP1B1 mRNA and protein levels (Table 9) [76]. These data suggest that carnosol may act via a mechanism involving the AhR to reduce PAH-induced carcinogenesis.

In a study by Alcaraz et al. (2013), treatment of B16F10 mouse melanoma cells with carnosol (20–40 µM) for 24–48 h prior to exposure to 10 Gy of X-rays exacerbated the effect of ionizing radiation as seen by an increase in cellular death by 34% (Table 9) [77]. Conversely, the same treatment in normal PNT2 prostate cells reduced radiation-induced cell death by 39%. [77]. Overall, these data suggest that carnosol may offer protection to healthy cells while rendering cancerous cells more susceptible to radiotherapy. The mechanisms underlying these radiosensitizing properties of carnosol have yet to be identified. 

Exposure of human melanoma A375 cells to varying dilutions (1:120 to 1:960) of crude rosemary extract led to dose- and time-dependent reductions in cell viability, metabolic activity, and ROS levels and increased G2/M phase cell cycle arrest [78]. Various chromatography and spectrometry techniques were used to determine the principal components of the crude rosemary extract with carnosol found in the crude extract at a concentration of 80.1 µg/mL (~242 µM) [78]. Treatment with purified carnosol (1–50 μM) for 24–72 h resulted in reduced cell viability, as determined with a trypan blue exclusion assay. A375 cells showed a 100% reduction in metabolic activity with 20 and 50 μM carnosol treatment after 72 h. Additionally, 15% and 90% reductions in metabolic activity of A375 cells were evident after 72 h of 1 µM and 5 µM carnosol treatment, respectively, as determined with an 3-(4,5-Dimethylthiazol-2-yl)-2,5-Diphenyltetrazolium Bromide (MTT )assay (Table 9) [78]. Of the 5 principal compounds identified in the crude rosemary extract, carnosol had the most pronounced effect, while apigenin and luteolin significantly reduced viability and scutellarin and rosmarinic acid treatments were the least effective when used alone [78]. In general, these data suggest that carnosol contributes significantly to the anticancer properties of rosemary extract.

In a study by Tong and Wu (2018), the exposure of human keratinocytes (HaCaT) to carnosol (10–30 μM) for 12 h produced a dose-dependent reduction in ultraviolet B (UVB)-induced ROS [79]. Furthermore, carnosol treatment alongside UVB exposure saw a reduction in phosphorylated H2AX and Chk1, markers of DNA breakage and damage, and a 50% reduction in cyclobutane pyrimidine dimers, a common UVB-induced DNA lesion, compared to cells only exposed to UVB [79]. Similarly, carnosol was able to reduce by 50% the transformation rate of HaCaT cells that were repeatedly exposed to UVB radiation [79]. Lastly, carnosol was found to reduce UVB-induced activation of the pro-survival NF-κB pathway. UVB exposure led to a reduction in IκB, the inhibitor of NF-κB, but carnosol treatment was able to partially protect IκB, leading to reduced phosphorylation (Ser276) of NF-κB as well as reduced activity (Table 9) [79]. Overall, these data suggest that carnosol may offer protection against UVB-induced damage.

### 2.11. Effects of Carnosol on Skin Cancer In Vivo

Experiments by Huang et al. (1994) showed that topical/skin application of carnosol inhibited 12-*O*-tetradecanoyl-phorbol-13-acetate (TPA)-induced tumour formation [80]. Carnosol at 0.1–1 µmol in 20 µL of acetone (5–50 mM) was able to significantly reduce TPA-induced ear inflammation with 1.5 mM inhibiting edema by 67%, and 50 mM inhibiting edema by 100%. Furthermore, 1–10 µmol of carnosol in 200 µL of acetone (10–50 mM) was able to reduce TPA-induced ornithine decarboxylase activity in mouse epidermis by 35–70% [80]. Additionally, carnosol treatment was able to reduce TPA-induced tumour formation. Tumour formation was initiated with DMBA and then promoted with TPA application twice weekly. Application of 10, 15, or 50 mM carnosol during TPA applications reduced the average number of skin tumours per mouse by 37, 64, and 77%, respectively (Table 10) [80]. These data ultimately suggest that skin damage leading to tumour formation can be reduced by topical application of carnosol. 

Similarly, Núñez et al. (2011) found reduced DMBA/TPA-induced papilloma formation in female IRC mice treated with carnosol [81]. Mice were initially treated with 390 nmol (100 µg) of DMBA to initiate papilloma formation and then 1.7 nmol (1 µg) of TPA in 0.1 mL acetone was applied twice weekly, one week after initiation to promote papilloma formation. Mice in the carnosol treatment group received application of 85 nmol carnosol in 0.1 mL of acetone (850 µM) one h prior to application of TPA. In the control group, mice started showing papillomata at 6 weeks and by 10 weeks all mice had developed papillomata [81]. Conversely, carnosol treatment slowed the development of papillomata as only 40% of mice had papillomata at weeks 13–14 and 80% of mice had papillomata at the conclusion of the experiment at 20 weeks. Furthermore, carnosol treatment reduced the average number of papillomata with the control group having an average of 9.3 papillomata/mouse at week 20, whereas the carnosol group only had 4.2 papillomata/mouse (Table 10) [81]. Overall, these data suggest that topical application of carnosol may protect against the skin damage that leads to tumour formation.

## 3. Conclusions

Cancer is characterized by the unregulated proliferation of cells, the inhibition of apoptosis, altered metabolism, tissue invasion and metastasis, and the dysregulation of cell signalling that ultimately leads to enhanced survival, growth, and tumour formation. The identification of compounds that can target these important cancer characteristics, without detrimentally affecting healthy normal cells/tissues is of the utmost urgency. Carnosol has been shown to possess antioxidant, anti-inflammatory, and antimicrobial properties and has been identified as a highly favored polyphenol for cancer prevention and treatment. The current review summarizes all existing in vitro and in vivo studies that examined the anticancer effects of carnosol.

Treatment of breast cancer, prostate cancer, and skin cancer cells with carnosol significantly reduced cell viability, colony formation, cell proliferation and induced G2/M cell cycle arrest and apoptosis. Administration of carnosol in mice prevented the formation of DNA adducts and decreased the number of DMBA-induced mammary adenocarcinomas. Additionally, carnosol attenuated PAH-induced carcinogenesis in lung cancer cells via reduced DNA adduct formation. Colon cancer cells treated with carnosol had reduced viability, increased apoptosis, increased pro-apoptotic BAX expression and decreased anti-apoptotic Bcl-2 expression. Carnosol inhibited c-Met, induced apoptosis and prevented migration and colony formation in pancreatic cancer cells. 

**Figure 2:**
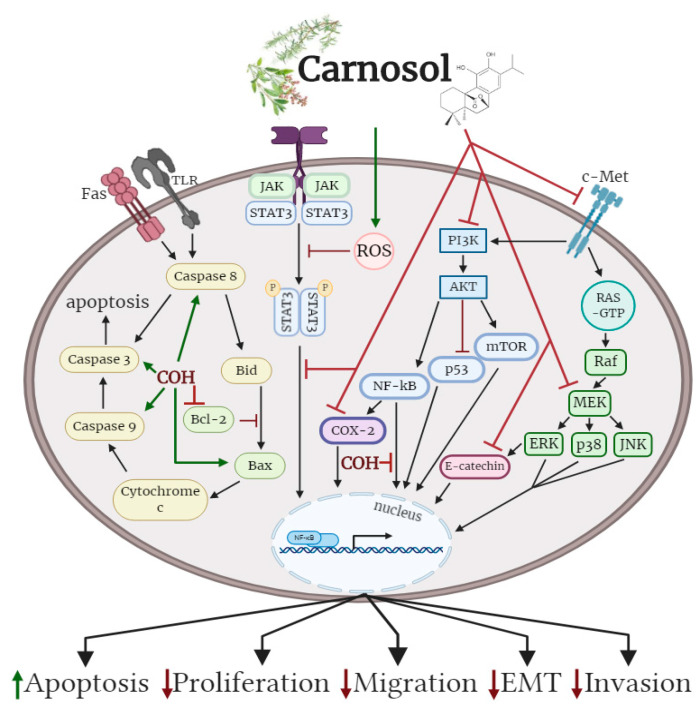
Effects of carnosol on cancer cell signalling molecules. Carnosol inhibited the phosphorylation/activation levels of ERK, p38, JNK, Akt, mTOR and COX-2. The nuclear translocation of NF-κB was prevented with carnosol, sequestering NF-κB in the cytosol. Pro-apoptotic caspase-3, -8, -9 and BAX protein levels were increased, while anti-apoptotic protein Bcl-2 was reduced with carnosol. The figure is based on the data of the studies [40,43,46,49,53,56,60,61,68,69,75,79] and created with BioRender.com. COH: carnosol.

Oral administration of carnosol reduced tumour growth, serum prostate specific antigen levels and decreased AR and ER-α protein expression in a xenograft model of prostate cancer. Treatment of differentiate glioblastoma cells with carnosol reduced cell viability and inhibited the processes that contribute to the aggressive and resistive nature of glioblastoma, including EMT and CSC formation. In addition, carnosol reduced TPA-induced ear inflammation, tumor formation and the average number of papillomata. The cellular effects of carnosol on the various subtypes of cancer are summarized in Figure 2.

Overall, these studies indicate that treatment with carnosol significantly attenuates key cancer characteristics, reducing cell viability, cell proliferation, colony formation, migration and promoting cancer cell apoptosis. However, more studies are required to fully understand the effects of carnosol in both cancerous and normal tissues. Notably, future in vitro studies should aim to expand our understanding of the effects of carnosol on cancers of the lung, colon, and pancreas as investigations involving cancer of these tissues are underrepresented in the literature. Additionally, more animal studies should be conducted including studies on the pharmacokinetics of carnosol to identify optimal dosage and routes of administration as well as xenograft studies to better understand the tumour reducing potential of carnosol to be used towards cancer treatment. Lastly, clinical studies are required to further explore the anticancer potential of carnosol in cancer patients.

## Figures and Tables

**Table 1:** Effects of carnosol on lung cancer in vitro.

Cell Line	Treatment	Effect	Reference
BEAS-2B (B[a]P-induced carcinogenesis)	0.6–6 µg/mL; 7–24 h	↓DNA adduct formation ↓CYP1A1 mRNA ↓CYP1A1 activity ↑GSTπ mRNA and protein ↑QR mRNA	[37]

**Table 2:** Effects of carnosol on colon cancer in vitro.

Cell Line	Treatment	Effect	Reference
HCT116	5–100 µM; 24–72 h	↓Viability ↑Apoptosis ↑c-Caspase-9 ↑c-Caspase-3 ↑c-PARP ↑p53 ↓Mdm2 ↓p-STAT3 ↓Cyclin-D1, -D2, and -D3 ↓Survivin ↓p-Jak2 ↓p-Src	[40]

**Table 3:** Effects of carnosol on breast cancer in vitro.

Cell Line	Treatment	Effect	Reference
184B5/HER	20–60 μM; 4.5 h	↓Tumorigenesis ↓COX-2 ↓AP-1 activity ↓PKC ↓ERK1/2 ↓P38 ↓JNK ↓PGE_2_ synthesis	[43]
MCF7	40 μM; 48 h	↓Cell viability IC_50_ 25.6 µM ↑Cytotoxic activity IC_50_ 82 µM ↓AR ↓ER-α	[44]
MCF7 MDA-MB-231	10–100 μM; 2–12 h	↓Proliferation IC_50_ 40 μM AMPK ↓CYP1A1 ↓AhR	[45]
MDA-MB-231	25–100 μM; 48 h	↓Cell Viability ↑ Apoptosis ↓Colony formation ↑Autophagy ↑ ROS generation ↑DNA damage ↑pERK1&2 ↑P21 ↑ Cleaved Parp Caspases 3,8,9	[46]
HBL-100 MDA-231 MDA-361 MDA-435 MCF-7	25–200 μM; 72 h	↓Cell Viability IC_50_ >50 μM ↓Cell adhesion ↓Growth	[47]
184-B5/HER	1–5 μM; 21 days	↓Colony formation IC_50_ 2.5 µM ↑G2 phase-specific cyclin B1 expression ↑G2/M phase arrest	[48]
MDA-MB-231 Hs578T MCF-7 T47D	25–100 μM; 24 h	↓Migration ↓Invasive potential MMP-9 ↓phosphorylated and total STAT3	[49]

**Table 4:** Effects of carnosol on breast cancer in vivo.

Model	Treatment	Effect	Reference
Female Sprague–Dawley rats (DMBA-induced tumorigenesis)	200 mg/kg; 5 days	↓DNA adduct formation ↓Mammary adenocarcinoma	[50]
Chick embryo (MDA-MB-231-GFP xenograph)	50–100 µM	↓Tumor mass ↓Metastases	[49]

**Table 5:** Effects of carnosol on pancreatic cancer in vitro.

Cell Line	Treatment	Effect	Reference
AsPC-1	0–75 µM; 8–48 h	↑Apoptosis ↓Proliferation ↑S-phase cell cycle arrest ↓Sphere formation ↓Colony formation ↓oct-4 ↓nanog	[53]

**Table 6:** Effects of carnosol on prostate cancer in vitro and in vivo.

Cell Line/Animal Model	Treatment	Effects	Reference
PC3	10–70 μM; 24–72 h	↑Apoptosis ↓Cell Viability ↑BAX ↓Bcl-2 ↑Caspase-7 and -8 ↓p21 (Waf1/Cip1) ↓Cyclins A, D1, D2 ↓CDK6 and CDK 2 ↓mTOR (Ser2448) ↓AMPK-α (Thr172) ↓p70 S6K ↑4E-BP1 ↑PTEN ↓PI3K (p85 and p110)	[56]
22 RV1 PCA LNCAP	20–40 μM; 48 h	↓Cell Viability ↓AR ↓ERα	[44]
LNCAP DU145	0.25–16 μM; 48 h	↑Apoptosis ↓Cell survival ↓Proliferation ↓GLi1 ↓Shh ↑Caspase 3 activity	[57]
Athymic nude mice implanted with 22RV1 cells	30 mg/kg; 28 days	↓Tumor growth ↓Serum prostate-specific antigen ↓AR ↓ER-α	[44]

**Table 7:** Effects of carnosol on leukemia in vitro.

Cell Line	Treatment	Effect	Reference
SEM RS4;11 MV4;11 REH Nalm-6	9–27 µM; 24 h	↑Apoptosis ↓Viability ↓Nuclear DNA ↑Depolarization of mitochondrial membranes ↓Bcl-2	[60]
ED	40 µM; 3–24 h	↑Apoptosis ↑Caspase-3 ↑Caspase-7 ↓Glutathione	[61]

**Table 8:** Effects of carnosol on brain cancer in vitro.

Cell Line	Treatment	Effect	Reference
U87MG U343MG T98G	0.1–100 µM; 24–72 h	↓Proliferation ↑Apoptosis ↓Cell migration ↑G2-cell cycle arrest ↑p53 protein ↓p53/MDM2 complex ↑p21 transcription ↑PUMA transcription ↑MDM2 transcription ↑BAX transcription ↓Bcl-2 transcription	[68]
U87MG U87MG-CSC U343MG-CSC T98G-CSC	10 nm–40 µM	↓CSC viability ↑CSC apoptosis ↑G2-cell cycle arrest ↓Mesenchymal phenotype ↓N-cadherin ↑E-cadherin ↓CD44 ↓Nanog ↓Oct4 ↓BMI1 ↓SOX2 ↓Nestin ↓OLIG2 ↑GFAP ↑p21 transcription ↑PUMA transcription ↑MDM2 transcription ↑BAX transcription ↓Bcl-2 transcription	[69]

**Table 9:** Effects of carnosol on skin cancer in vitro.

Cell Line	Treatment	Effect	Reference
B16/F10	1.25–20 μM; 6–24 h	↓Migration ↓Invasion ↓Colony formation IC_50_ 5 µM ↓MMP-9 ↓ERK1/2 ↓AKT ↓p38 ↓JNK ↓NF-κB ↓c-Jun	[75]
HaCaT	1–10 μM; 2 h	↓CYP1A1 ↓CYP1B1	[76]
B16F10	20–40 µM; 24–48 h	↑Radiosensitivity ↓Cell survival	[77]
A375 melanoma	1–50 μM; 24–72 h	↓Cell Viability	[78]
HaCaT	10–30 μM; 12 h	↓ROS ↓DNA damage ↓γH2AX ↓p-Chk1 ↓CDP ↓Transformation ↑IκB ↓NF-κB	[79]

**Table 10:** Effects of carnosol on skin cancer in vivo.

Model	Treatment	Effect	Reference
Female CD-1 mice (DMBA and TPA-induced tumorigenesis)	0.1–1 µmol in 20 µL acetone (5–50 mM) 1–10 µmol in 0.2 mL acetone (10–50 mM)	↓Tumorigenesis ↓ Ornithine decarboxylase activity ↓TPA-induced ear inflammation	[80]
Female ICR mice (DMBA and TPA-induced tumorigenesis)	85 nmol in 0.1 mL acetone (850 µM)	↓Tumorigenesis ↓Rate of papilloma formation ↓Number of papillomata	[81]
